# Supplementary material for: Parameterized resetting model captures dose-dependent entrainment of the mouse circadian clock
Source: Nat Commun. 2025 Feb 6;16:1421. doi: 10.1038/s41467-025-56792-z (PMC11802799; doi:10.1038/s41467-025-56792-z)
Supplement: Supplementary file 1 — Supplementary Information [file 41467_2025_56792_MOESM1_ESM.pdf]

Parameterized resetting model captures dose-dependent entrainment of the mouse circadian clock

Supplementary Note 1

Supplementary Figure 1-13

Supplementary Note 1. Simulations for SR in desynchronized oscillator population.

The SR parameters can be not only calculated from the PRC, but also experimentally measured as the response of the desynchronized oscillator population. For the Stuart-Landau equation (35), including a circular limit cycle as shown in Fig. 1a, the SR parameters obtained from the PRC and the population response, respectively, agreed when the oscillators sufficiently recovered from the deviation from the limit cycle after stimulation (Supplementary Fig. 6). However, if the relaxation to the limit cycle was slow, the SR amplitude was overestimated. In this case, the error can be reduced by normalizing the amplitude at the maximum response shown in Supplementary Fig. 6c.

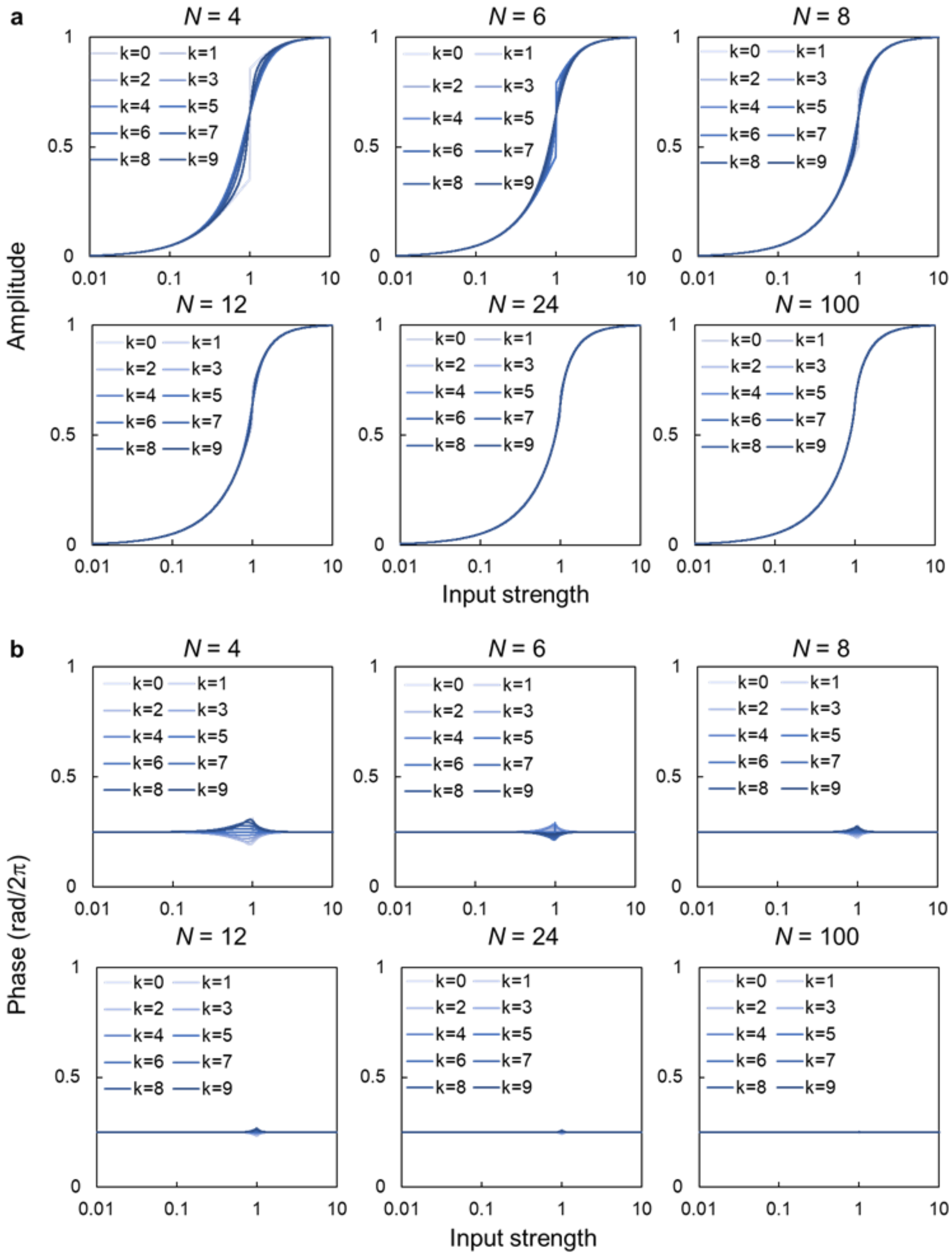

Supplementary Fig. 1. Effects of sample size in calculating SR parameters from PRC

a. Relationship between the sample size and SR amplitude.

b. Relationship between the sample size and SR phase.

SR amplitudes and phases were obtained from Eq. (4) for $\theta_j = 2\pi(j + k/10)/N\ (0 \leq j < N)$ for the PRC obtained from Eq. (1). k represents the shift of the starting point of equally divided points.

SR, singularity response; PRC, phase response curve

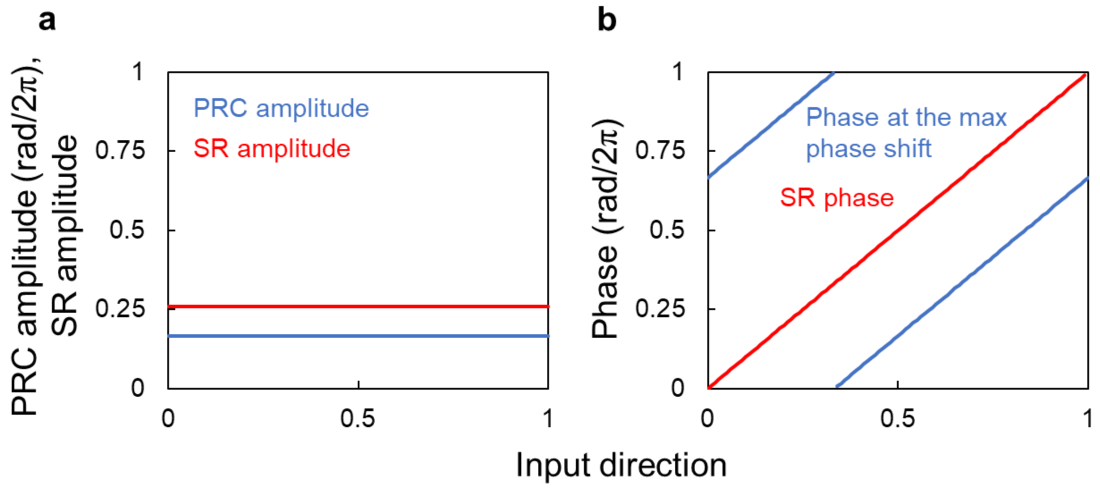

Supplementary Fig. 2. Changes in SR parameters in response to changes in input direction

a. Changes in SR amplitude and the maximum phase response in the PRC are plotted against the input direction, represented by the red arrow in Fig. 1a.

b. Changes in SR phase and the phase at which the maximum phase response is observed in the PRC are plotted against the input direction.

SR, singularity response; PRC, phase response curve

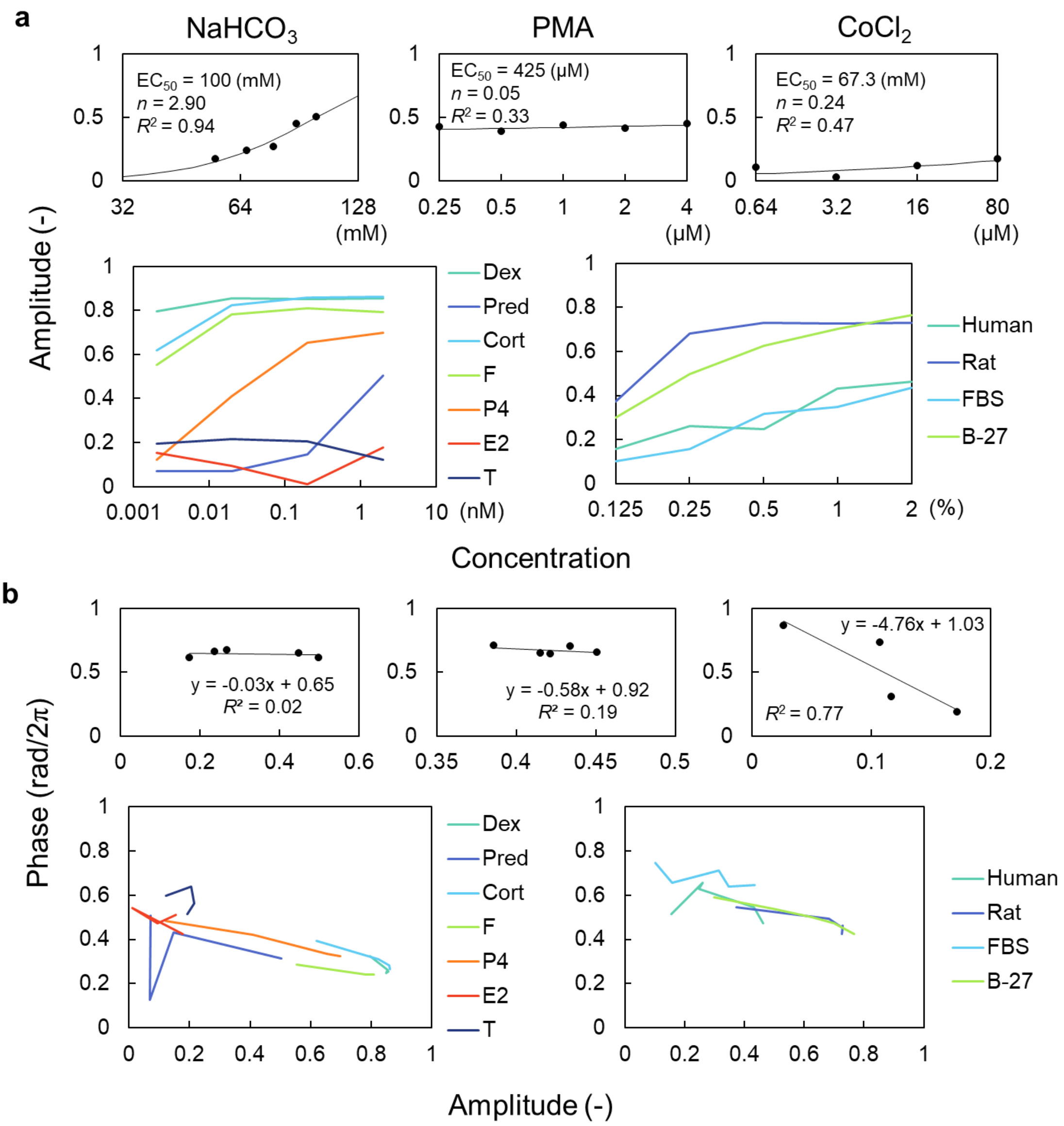

Supplementary Fig. 3. Dose-response curves of SR parameters for various stimuli measured in previous studies

a. Dose-dependent SR amplitudes for some types of resetting reagents.

b. SR phase for some types of resetting reagents. Each abbreviation denotes $NaHCO_3$: sodium hydrogen carbonate, PMA: phorbol 12-myristate 13-acetate, $CoCl_2$: cobalt chloride, Dex: dexamethasone, Pred: prednisone, Cort: corticosterone, F: hydrocortisone, P4: progesterone, E2: β-estradiol, T: testosterone, Human: human serum, Rat: rat serum, FBS: fetal bovine serum. B-27 was used as a supplement in the culture medium.

SR, singularity response

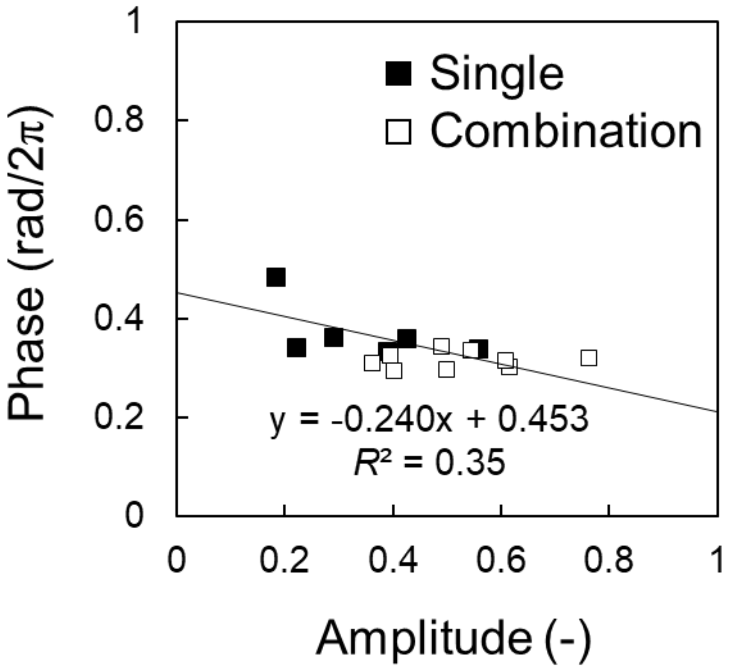

Supplementary Fig. 4. Relationship between SR amplitude and phase for mixed stimuli (hydrocortisone and corticosterone)

Filled squares indicate responses to a single stimulus of hydrocortisone or corticosterone, and blank squares indicate responses to a mixture of these stimuli. The solid line represents an approximate fit to the data points, and the corresponding equation is shown in the illustration above.

SR, singularity response

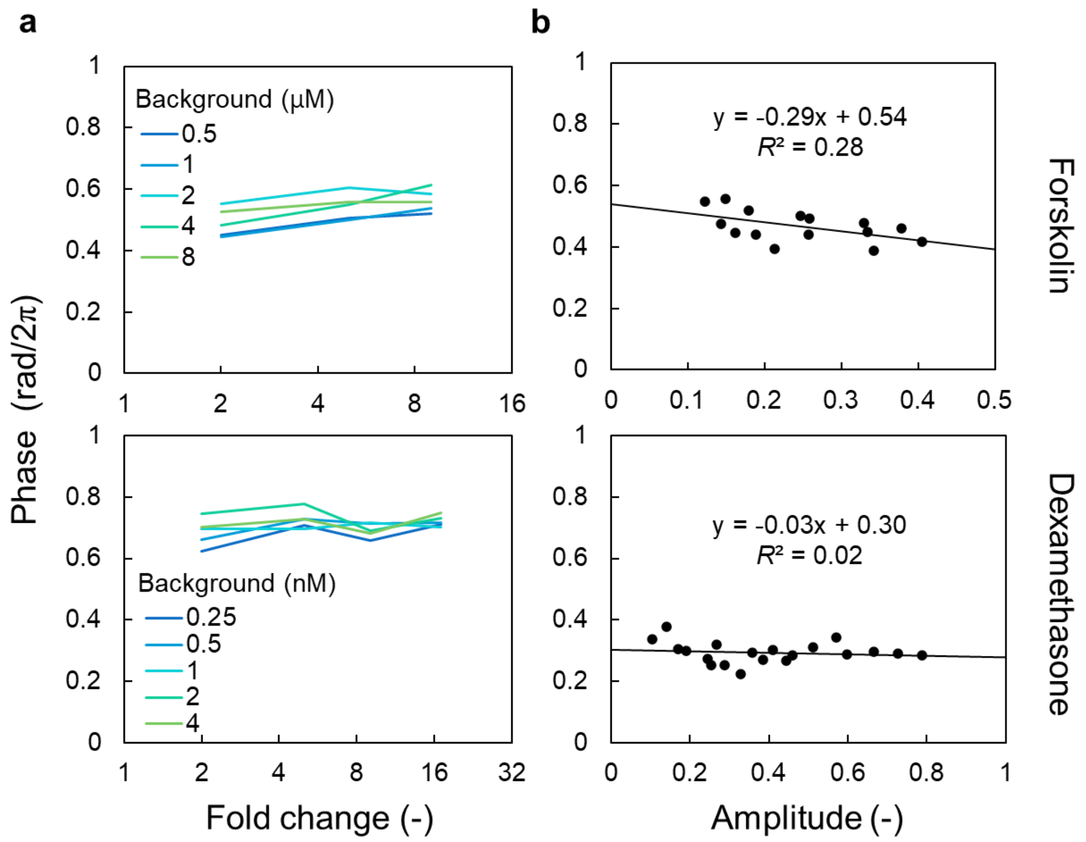

Supplementary Fig. 5. Background effect on SR phase parameters

a. SR phase for fold-change in stimulus strength (forskolin and dexamethasone) relative to the background concentration.

b. Amplitude and phase relationships under background conditions similar to those shown in **a**.

SR, singularity response

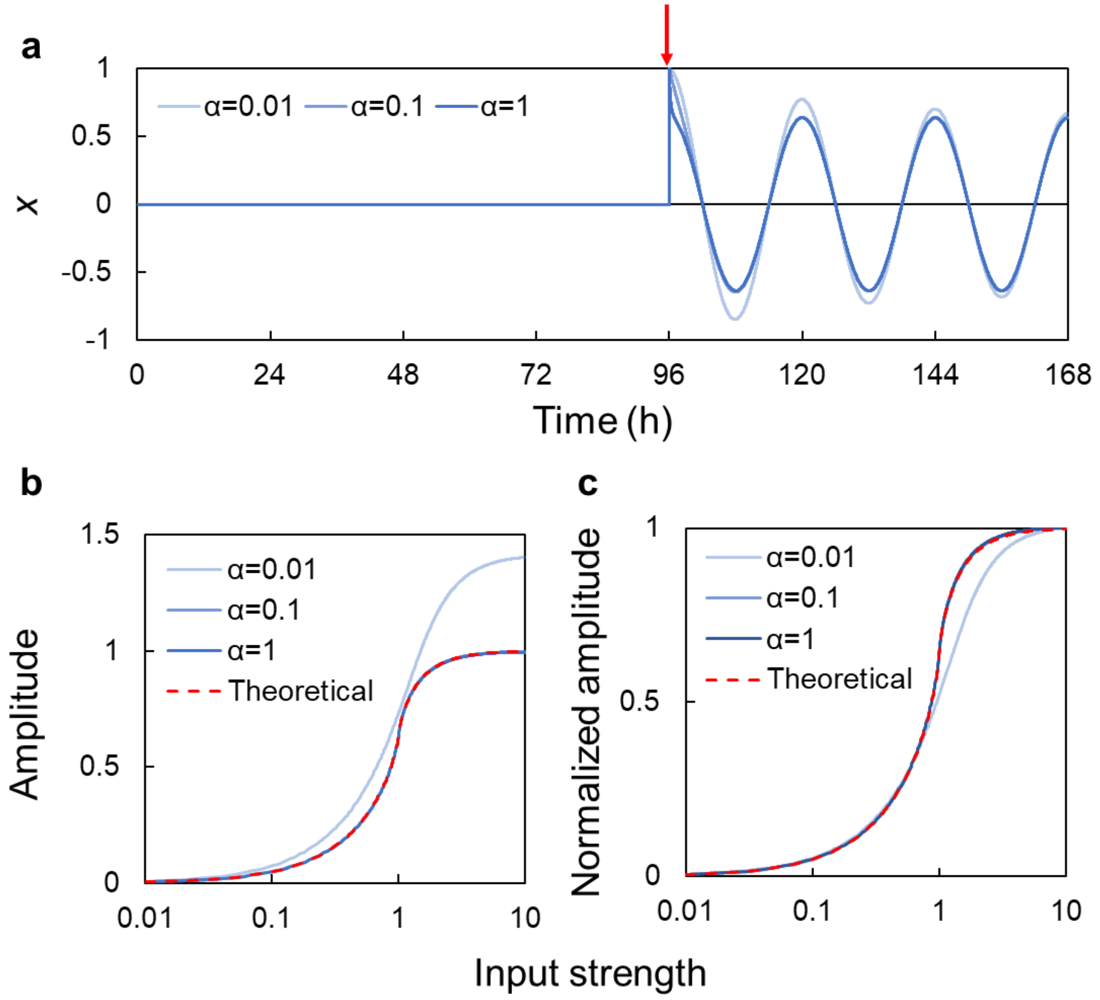

Supplementary Fig. 6. SR with slow recovery to a limit cycle

a. Response of oscillator population with slow recovery to a limit cycle. α indicates the speed of recovery to the limit cycle. The arrow indicates the time of stimulation.

b. SR amplitude with slow recovery to the limit cycle. "Theoretical" is the value obtained by calculating SR from PRC of Eq. (1).

c. Normalized SR amplitude. Each line is the value of Supplementary Fig. 3b divided by the maximum value in each condition.

SR, singularity response; PRC, phase response curve

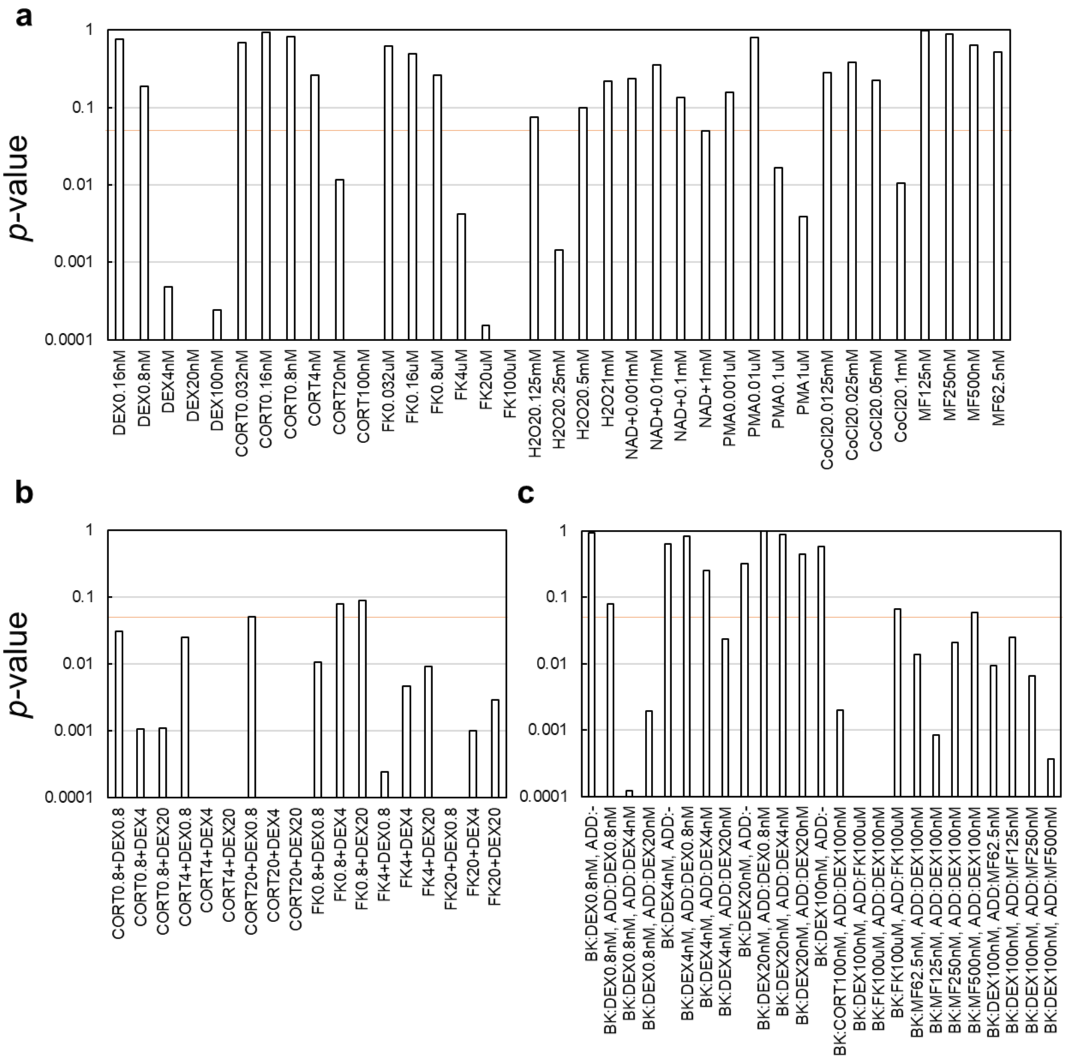

Supplementary Fig. 7. Comparison of SR amplitudes between stimuli and controls in cell culture

This figure compares the SR amplitudes observed for different experimental conditions with control conditions (two-tailed *t*-test). Statistical significance is indicated for each comparison. The orange lines represent the threshold for statistical significance (p=0.05).

a. Single stimulus: This panel shows the SR amplitudes for each stimulus compared with the control condition.

b. Mixture of stimuli: This panel shows the SR amplitudes observed for combined stimuli compared with the control condition.

c. Single stimulus with background: This panel shows the SR amplitudes observed for a single stimulus with a pre-treatment as the background stimulus, compared with the control condition.

SR, singularity response

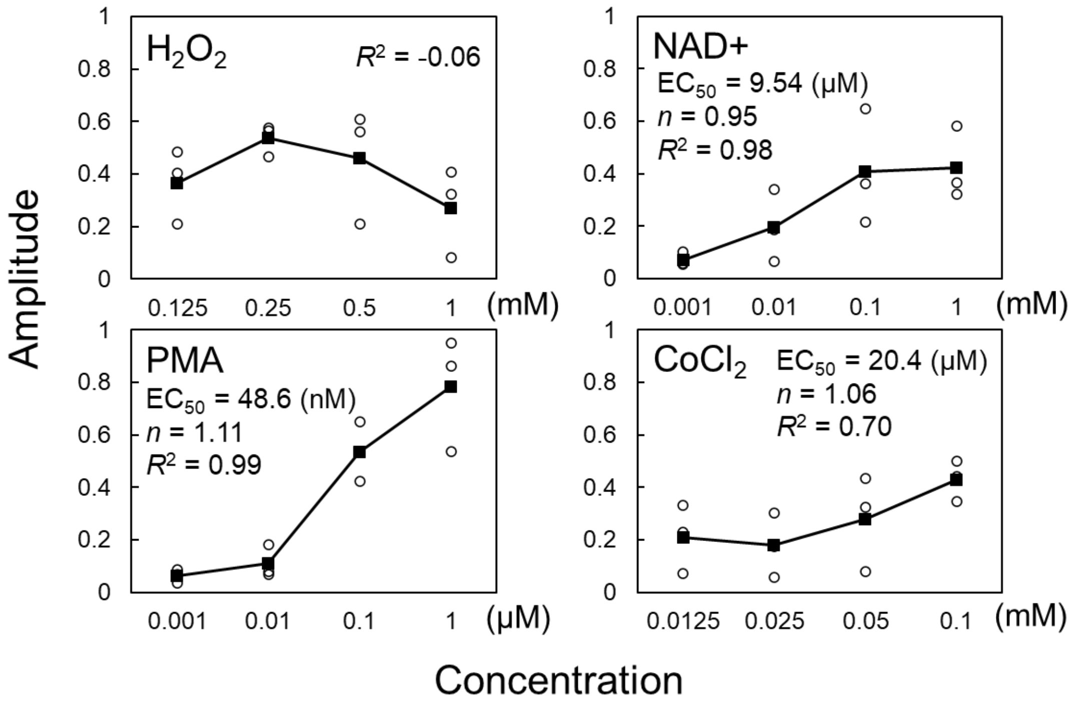

Supplementary Fig. 8. Dose-response curves of SR amplitude for various stimuli in PER2::LUC MEFs

Blank circles indicate individual data, and filled squares indicate mean values.

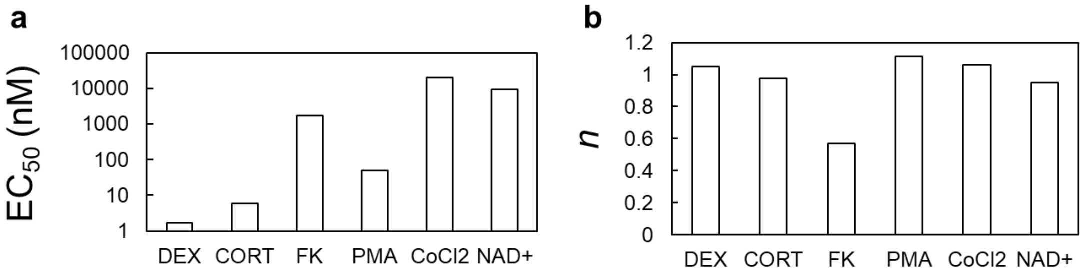

Supplementary Fig. 9. Parameters derived from dose-response curves for various stimuli in PER2::LUC MEFs

a. EC_{50} of dose-response curves for the indicated stimuli.

b. Slope parameter *n* of dose-response curves.

MEF, mouse embryonic fibroblast

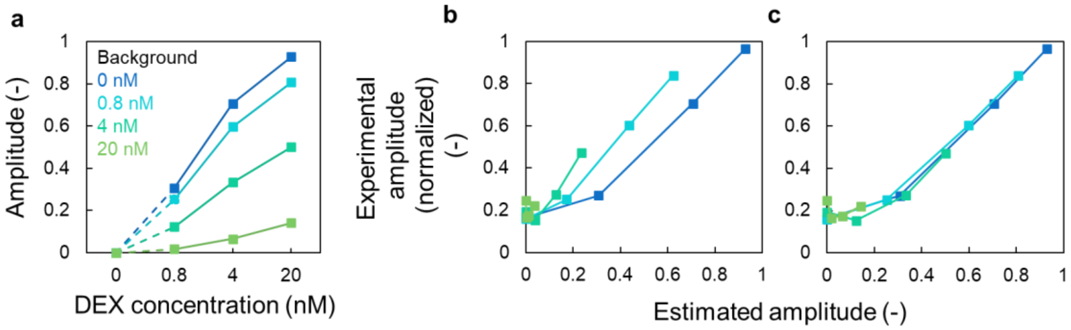

Supplementary Fig. 10. Predicted amplitudes when background concentration is reduced by 1/3 in PER2::LUC MEFs

a. Predicted amplitude for dexamethasone (DEX) stimulation with a dexamethasone background when the background concentration was reduced by 1/3 at the time of stimulation.

b, c. Relationship between measured SR amplitude in experiments and predicted amplitude when background concentrations did not change (b) and decrease to 1/3 concentration (c). The experimental values were normalized by dividing them by the SR amplitude for 100 nM DEX, which was the maximum response to DEX treatment.

SR, singularity response; DEX, dexamethasone

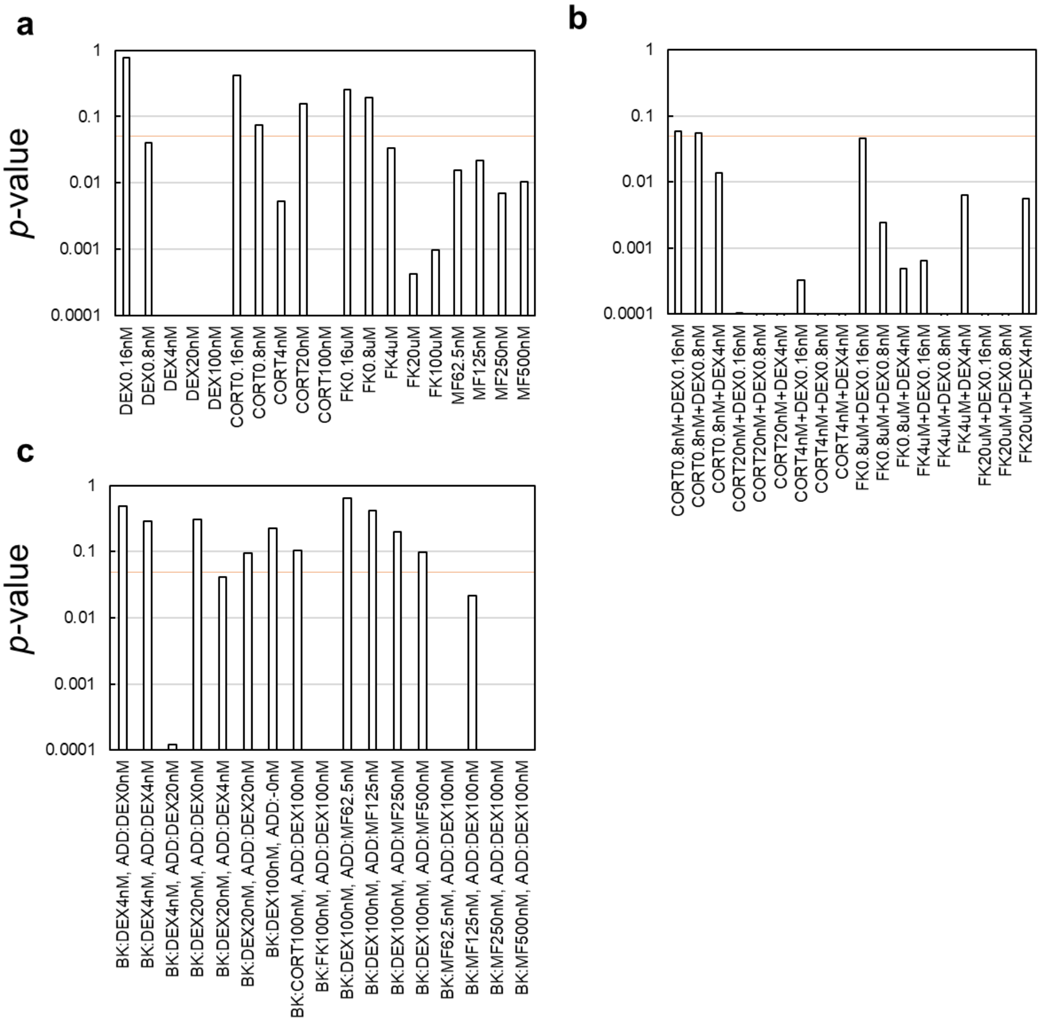

Supplementary Fig. 11. Comparison of SR amplitudes between each stimulus and control condition in lung tissues

This figure compares the SR amplitudes observed for different experimental conditions in lung tissue with control conditions (two-tailed *t*-test). Statistical significance is indicated for each comparison. The orange lines represent the threshold for statistical significance (p=0.05).

a. Single stimulus: This panel shows the SR amplitudes for each stimulus compared with the control condition.

b. Mixture of stimuli: This panel shows the SR amplitudes observed for combined stimuli compared with the control condition.

c. Single stimulus with background**:** This panel shows the SR amplitudes observed for a single stimulus with a pre-treatment as the background stimulus, compared with the control condition.

SR, singularity response

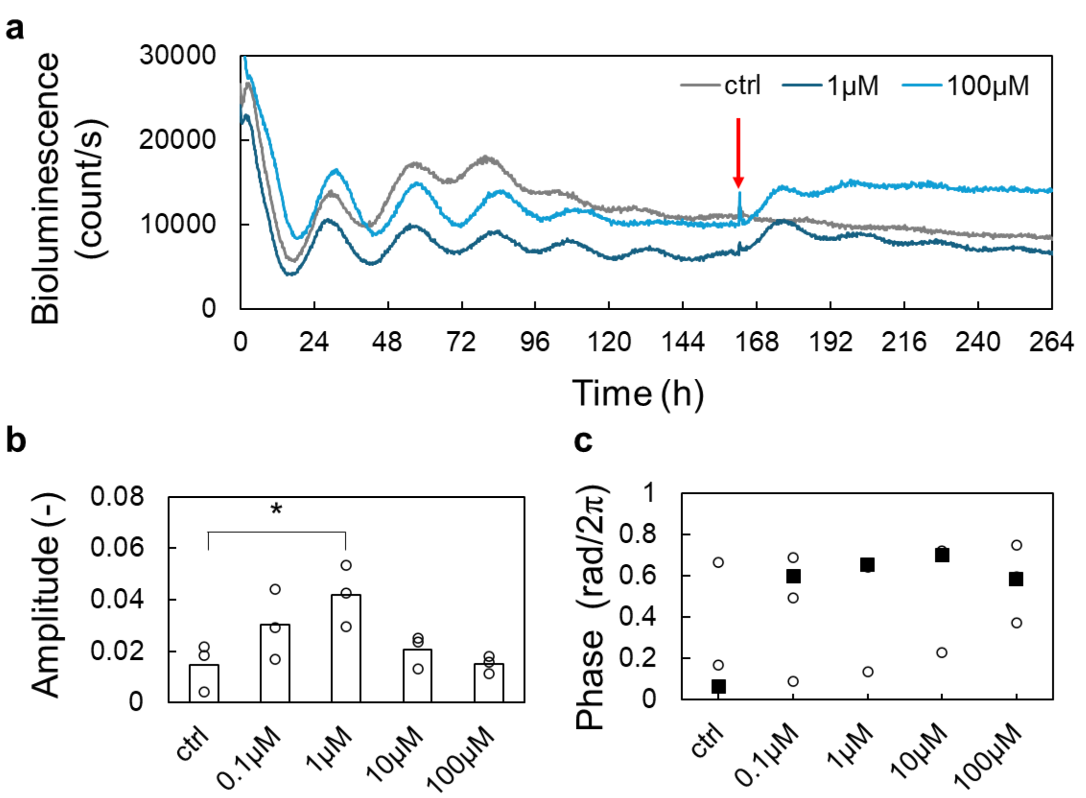

Supplementary Fig. 12. SR to forskolin in SCN.

a. Representative bioluminescence data for SR to forskolin stimulation. The arrow indicates the time of stimulation.

b. SR amplitude to forskolin for a series of concentration in SCN (*: $p<0.05$; two-tailed *t*-test).

c. SR phase to forskolin for a series of concentration in SCN.

SR, singularity response; SCN, suprachiasmatic nucleus

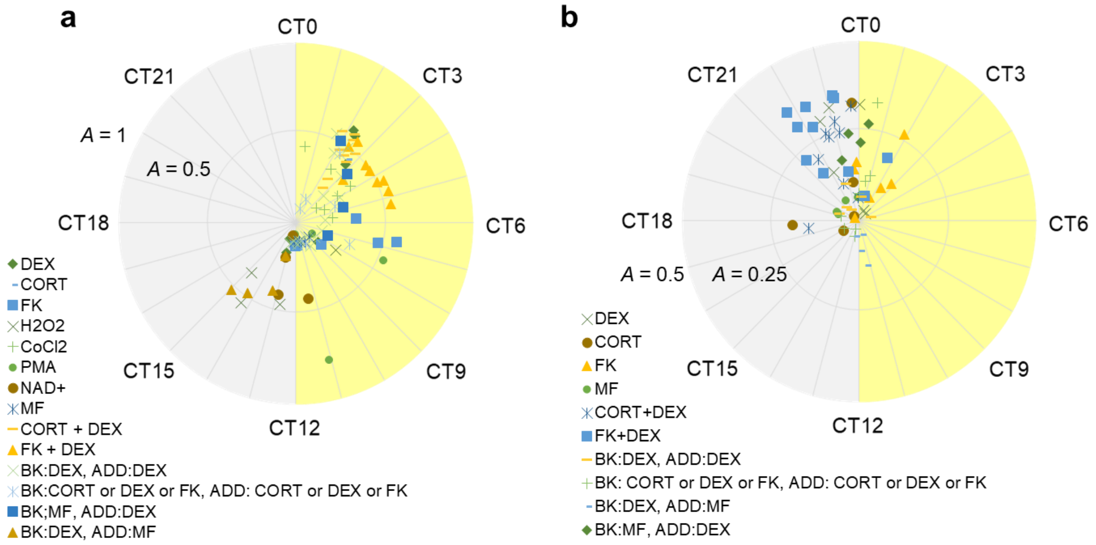

Supplementary Fig. 13. SR parameters for various stimuli

a. SR parameter in cultured MEFs.

b. SR parameters in the lung slice cultures. CT12 was defined as the peak of the *PER2*::*LUC* rhythm. The scale of SR amplitude *A* is shown.

MEF, mouse embryonic fibroblast; SR, singularity response
