# Supplementary material for: Parameterized resetting model captures dose-dependent entrainment of the mouse circadian clock
Source: Nat Commun. 2025 Feb 6;16:1421. doi: 10.1038/s41467-025-56792-z (PMC11802799; doi:10.1038/s41467-025-56792-z)
Supplement: Supplementary file 2 — Reporting Summary [file 41467_2025_56792_MOESM2_ESM.pdf]

nature portfolio

Corresponding author(s): Arisa Hirano

Last updated by author(s): Jan 17, 2025

Reporting Summary

Nature Portfolio wishes to improve the reproducibility of the work that we publish. This form provides structure for consistency and transparency in reporting. For further information on Nature Portfolio policies, see our Editorial Policies and the Editorial Policy Checklist.

Statistics

For all statistical analyses, confirm that the following items are present in the figure legend, table legend, main text, or Methods section.

n/a	Confirmed	
☐	☒	The exact sample size (*n*) for each experimental group/condition, given as a discrete number and unit of measurement
☐	☒	A statement on whether measurements were taken from distinct samples or whether the same sample was measured repeatedly
☐	☒	The statistical test(s) used AND whether they are one- or two-sided<br>*Only common tests should be described solely by name; describe more complex techniques in the Methods section.*
☒	☐	A description of all covariates tested
☒	☐	A description of any assumptions or corrections, such as tests of normality and adjustment for multiple comparisons
☐	☒	A full description of the statistical parameters including central tendency (e.g. means) or other basic estimates (e.g. regression coefficient) AND variation (e.g. standard deviation) or associated estimates of uncertainty (e.g. confidence intervals)
☐	☒	For null hypothesis testing, the test statistic (e.g. *F*, *t*, *r*) with confidence intervals, effect sizes, degrees of freedom and *P* value noted<br>*Give P values as exact values whenever suitable.*
☒	☐	For Bayesian analysis, information on the choice of priors and Markov chain Monte Carlo settings
☒	☐	For hierarchical and complex designs, identification of the appropriate level for tests and full reporting of outcomes
☒	☐	Estimates of effect sizes (e.g. Cohen's *d*, Pearson's *r*), indicating how they were calculated

Our web collection on statistics for biologists contains articles on many of the points above.

Software and code

Policy information about availability of computer code

Data collection	We used the software attached with the measurement device, Kronos HT (ATTO, Japan), for the bioluminescence measurements.
Data analysis	Python (version 3.11.1) with the library lmfit (version 1.1.0) was used for the calculation of phase and amplitude.<br>Fitting of dose-response curve was performed using the solver function of Microsoft Excel (version 2403 build 16.0.17425.20124).

For manuscripts utilizing custom algorithms or software that are central to the research but not yet described in published literature, software must be made available to editors and reviewers. We strongly encourage code deposition in a community repository (e.g. GitHub). See the Nature Portfolio guidelines for submitting code & software for further information.

Data

Policy information about availability of data

All manuscripts must include a data availability statement. This statement should provide the following information, where applicable:

- Accession codes, unique identifiers, or web links for publicly available datasets
- A description of any restrictions on data availability
- For clinical datasets or third party data, please ensure that the statement adheres to our policy

All data needed to evaluate the conclusions of this study are presented in the manuscript and/or Supplementary Information. The raw bioluminescence data generated in this study are provided in the Source Data file.

Research involving human participants, their data, or biological material

Policy information about studies with human participants or human data. See also policy information about sex, gender (identity/presentation), and sexual orientation and race, ethnicity and racism.

Reporting on sex and gender	NA
Reporting on race, ethnicity, or other socially relevant groupings	NA
Population characteristics	NA
Recruitment	NA
Ethics oversight	NA

Note that full information on the approval of the study protocol must also be provided in the manuscript.

Field-specific reporting

Please select the one below that is the best fit for your research. If you are not sure, read the appropriate sections before making your selection.

☒ Life sciences ☐ Behavioural & social sciences ☐ Ecological, evolutionary & environmental sciences

For a reference copy of the document with all sections, see nature.com/documents/nr-reporting-summary-flat.pdf

Life sciences study design

All studies must disclose on these points even when the disclosure is negative.

Sample size	Based on the results of previous SR measurements (Masuda, et al., 2023), the sample size was set at 3 for all sample sizes, and 5 for tissue cultures with large data variability, without sample size calculation.<br>These sample sizes were sufficient to assess differences from control conditions at the maximum concentration of each stimulus, and to draw dose-response curves because R^2 value exceeded 0.9 for most conditions.
Data exclusions	Data with no bioluminescence (measured values around 0) were excluded from the analysis.
Replication	The experiments were performed at two times for SCN slice and three or more times for the other experiments to verify the reproducibility. Reproducibility was observed between experiments and between cells and tissues under conditions of sufficiently strong stimulation.
Randomization	Randomization is not relevant because each experiment measures samples obtained from the same dishes for cell culture and the same individuals for peripheral tissue slice at the same time. The selection of individuals for each experiment was done randomly, except for sex. For SCN slice, individuals were randomly selected and randomly allocated into the experimental groups.
Blinding	Blinding is not relevant because the procedure of experiments and analysis is fixed regardless of the pre-experimental condition.

Reporting for specific materials, systems and methods

We require information from authors about some types of materials, experimental systems and methods used in many studies. Here, indicate whether each material, system or method listed is relevant to your study. If you are not sure if a list item applies to your research, read the appropriate section before selecting a response.

Materials & experimental systems

n/a	Involved in the study	
☒	☐	Antibodies
☐	☒	Eukaryotic cell lines
☒	☐	Palaeontology and archaeology
☐	☒	Animals and other organisms
☒	☐	Clinical data
☒	☐	Dual use research of concern
☒	☐	Plants

Methods

n/a	Involved in the study	
☒	☐	ChIP-seq
☒	☐	Flow cytometry
☒	☐	MRI-based neuroimaging

Eukaryotic cell lines

Policy information about cell lines and Sex and Gender in Research

Cell line source(s)	PER2::LUC MEFs were established from PER2::LUC mice (Jackson Laboratory, strain #006852) originate from mice generated by Yoo et al. (Proc Natl Acad Sci U S A., 2004,101:5339-46).
Authentication	The cell lines were not authenticated.
Mycoplasma contamination	The cell lines were not tested for mycoplasma contamination.
Commonly misidentified lines (See ICLAC register)	No commonly misidentified cell lines were used.

Animals and other research organisms

Policy information about studies involving animals; ARRIVE guidelines recommended for reporting animal research, and Sex and Gender in Research

Laboratory animals	Male C57BL/6J PER2::LUC mice aged 3-8 mouthes were used for peripheral tissues and male and female mice aged 7-24weeks for SCN. PER2::LUC mice (Jackson Laboratory, strain #006852) originate from mice generated by Yoo et al. (Proc Natl Acad Sci U S A., 2004,101(15):5339-46). Animals were maintained under a 12:12h light/dark cycle at 23.5 ± 2.0 °C and 50.0 ± 10.0 % humidity. Food and water were available ad libitum.
Wild animals	This study did not involve wild animals.
Reporting on sex	For peripheral tissues, the findings in the animal experiments can apply to only male mice; sex difference was not considered.<br>For SCN slice, male and female mice were randomly allocated into the experiments and sex-based analysis was not performed due to lack of sufficient sample size.
Field-collected samples	This study did not involve samples collected from the field.
Ethics oversight	All experiments involving animals were approved by the Animal Experiment and Use Committee of the University of Tsukuba and were therefore in accordance with NIH guidelines.

Note that full information on the approval of the study protocol must also be provided in the manuscript.

Plants

Seed stocks	*Report on the source of all seed stocks or other plant material used. If applicable, state the seed stock centre and catalogue number. If plant specimens were collected from the field, describe the collection location, date and sampling procedures.*
Novel plant genotypes	*Describe the methods by which all novel plant genotypes were produced. This includes those generated by transgenic approaches, gene editing, chemical/radiation-based mutagenesis and hybridization. For transgenic lines, describe the transformation method, the number of independent lines analyzed and the generation upon which experiments were performed. For gene-edited lines, describe the editor used, the endogenous sequence targeted for editing, the targeting guide RNA sequence (if applicable) and how the editor was applied.*
Authentication	*Describe any authentication procedures for each seed stock used or novel genotype generated. Describe any experiments used to assess the effect of a mutation and, where applicable, how potential secondary effects (e.g. second site T-DNA insertions, mosiacism, off-target gene editing) were examined.*
